# Supplementary material for: Nanowarming and ice-free cryopreservation of large sized, intact porcine articular cartilage
Source: Commun Biol. 2023 Feb 24;6:220. doi: 10.1038/s42003-023-04577-9 (PMC9958003; doi:10.1038/s42003-023-04577-9)
Supplement: Supplementary file 2 — Supplementary Information [file 42003_2023_4577_MOESM2_ESM.pdf]

SUPPLEMENTARY INFORMATION

Nanowarming and ice-free cryopreservation of large sized, intact porcine articular cartilage

Peng Chen, Shangping Wang, Zhenzhen Chen, Pengling Ren, R. Glenn Hepfer, Elizabeth D. Greene, Lia H. Campbell, Kristi L. Helke, Xingju Nie, Jens H. Jensen, Cherice Hill, Yongren Wu, Kelvin G.M. Brockbank, Hai Yao

Content

Supplementary Figure 1-6

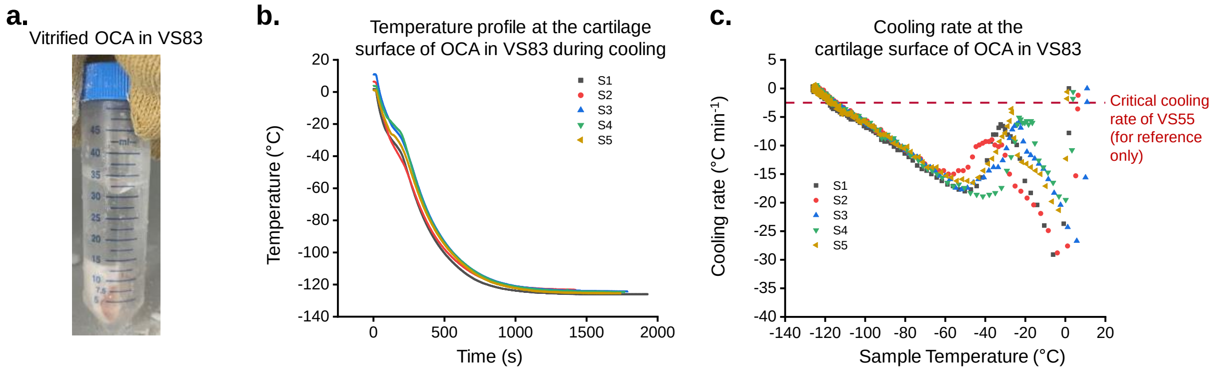

Supplementary Figure 1: Successful OCA vitrification. a A photograph of vitrified OCA in VS83. The tube containing the OCA and VS83 is clear, and no cracks or ice formation are observed, indicating successful OCA vitrification. **b** Temperature-time curve recorded at the center of the cartilage surface of OCA in VS83 during the cooling process. n = 5 independent samples. **c** Cooling rate-temperature curve derived from the data presented in **b**. The red dashed line showed the critical cooling rate of VS55. The critical cooling rate of VS55 shown here is only for reference. The actual critical cooling rate in VS83 is expected to be above the red dashed lineless (the VS55 critical cooling rate) due to the higher CPA concentration in VS83. n = 5 independent samples.

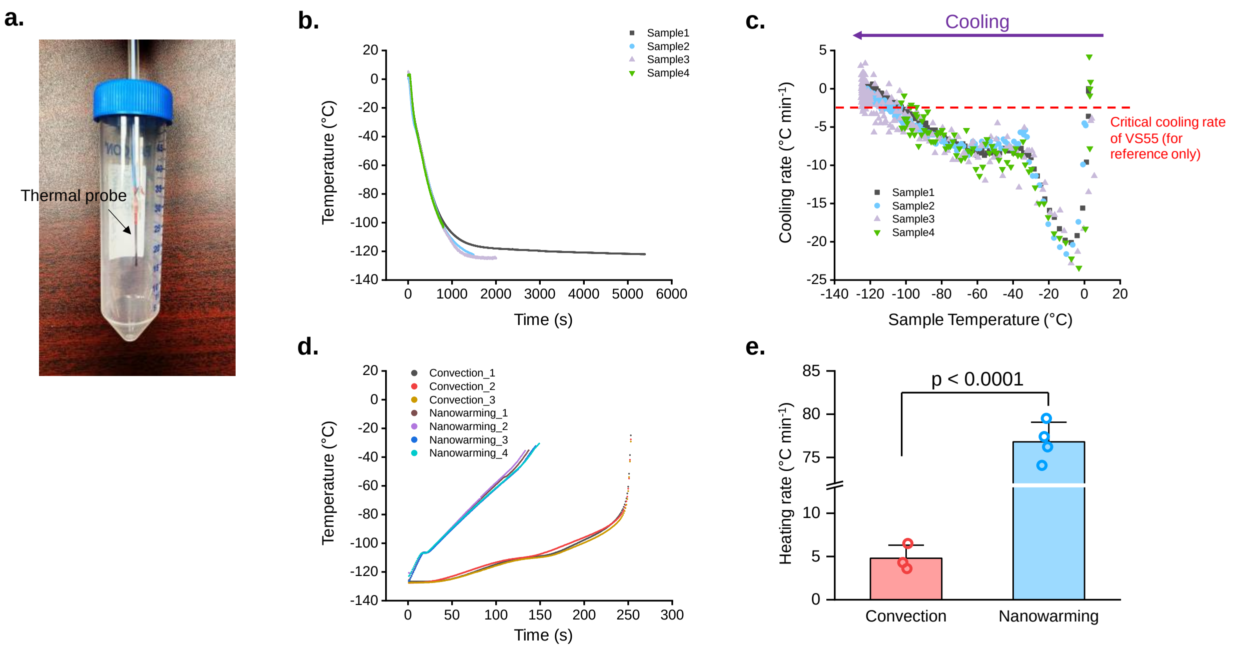

Supplementary Figure 2: Cooling and heating rate measurement in VS83 solutions. a Tube with fiber optical thermal probe for experimental temperature measurement. **b** Temperature-time curve recorded during the cooling process. n = 4 independent samples. **c** Cooling rate-temperature curve derived from the data presented in **b**. The red dashed line showed the critical cooling rate of VS55. The critical cooling rate of VS55 shown here is only for reference. The actual critical cooling rate in VS83 is expected to be above the red dashed lineless (the VS55 critical cooling rate) due to the higher CPA concentration in VS83. n = 4 independent samples. **d** Temperature-time curve recorded during the warming process. n = 3 independent samples for the convection group and n = 4 independent samples for the nanowarming group. **e** Heating rate measurement derived from the data presented in **d**. n = 3 independent samples for the convection group and n = 4 independent samples for the nanowarming group. p-value was determined with a two-sided t-test. All data depict mean ± standard deviation.

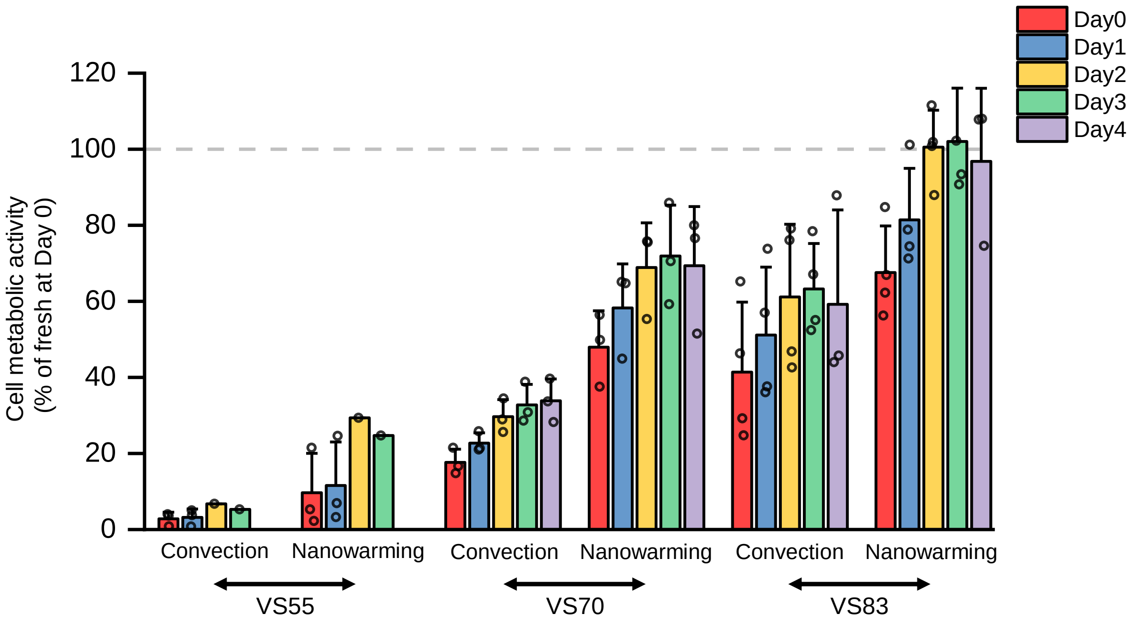

Supplementary Figure 3: Cell metabolic activities measured with cartilage preserved with VS55, VS70, and VS83. n = 3 independent samples for convection and nanowarming with VS55. n = 3 independent samples for convection and nanowarming with VS70. n = 4 independent samples for convection and nanowarming with VS83. The grey dashed line indicates 100% recovery, reaching the level of cell metabolic activity of fresh cartilage. Cell metabolic activities were only monitored for the first three days of tissue culture in the VS55 groups as cell metabolic activities in VS55 groups were much smaller than those measured from VS70 and VS83. All data depict mean ± standard deviation.

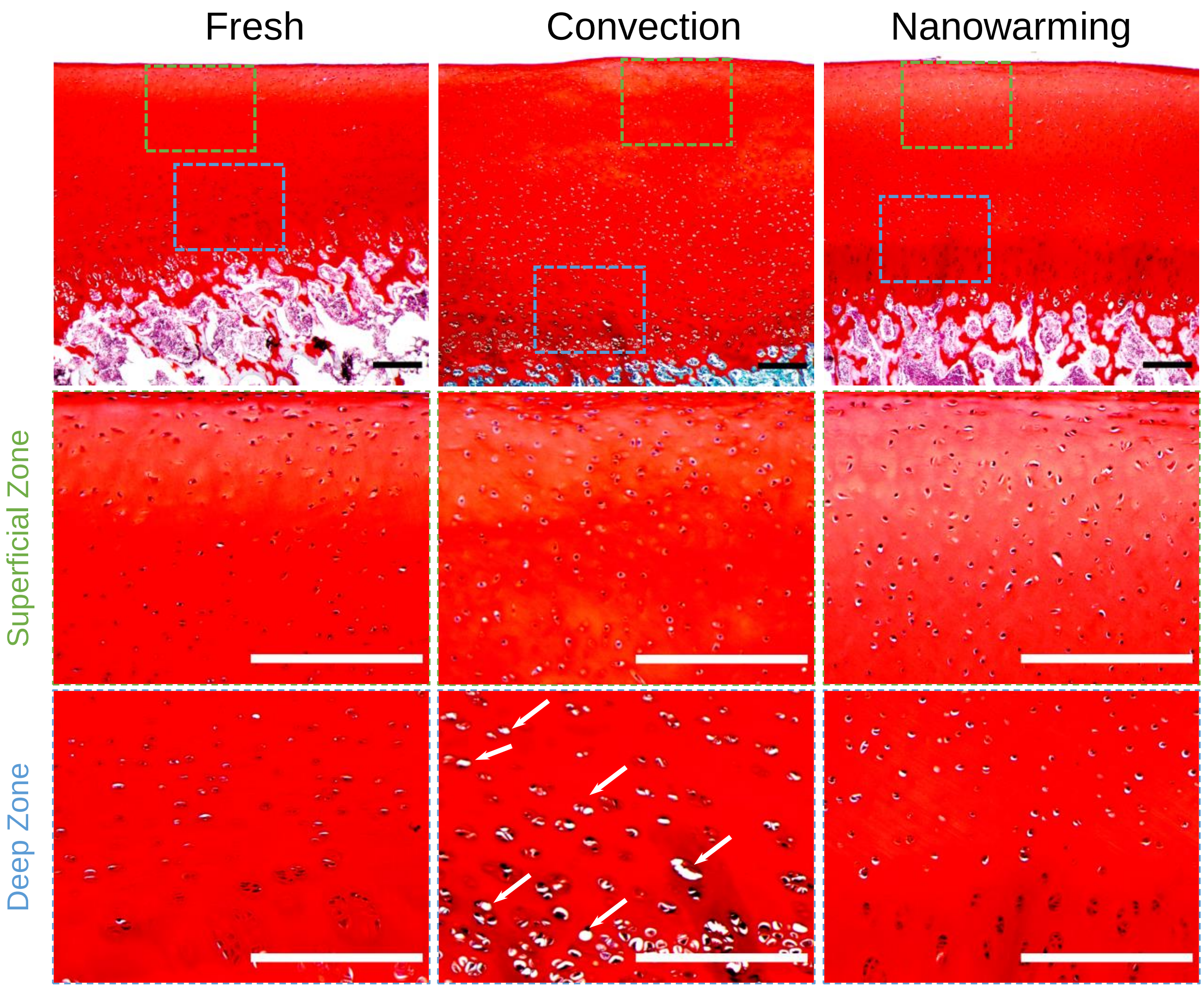

Supplementary Figure 4: Safranin O staining of articular cartilage from fresh, convection, and nanowarming groups. The zoomed-in superficial zone and deep zone were highlighted in the first row of images with green and blue dashed boxes, respectively. The white arrows highlight locations with white space, indicating possible ice formation. All scale bars represent 200 μm.

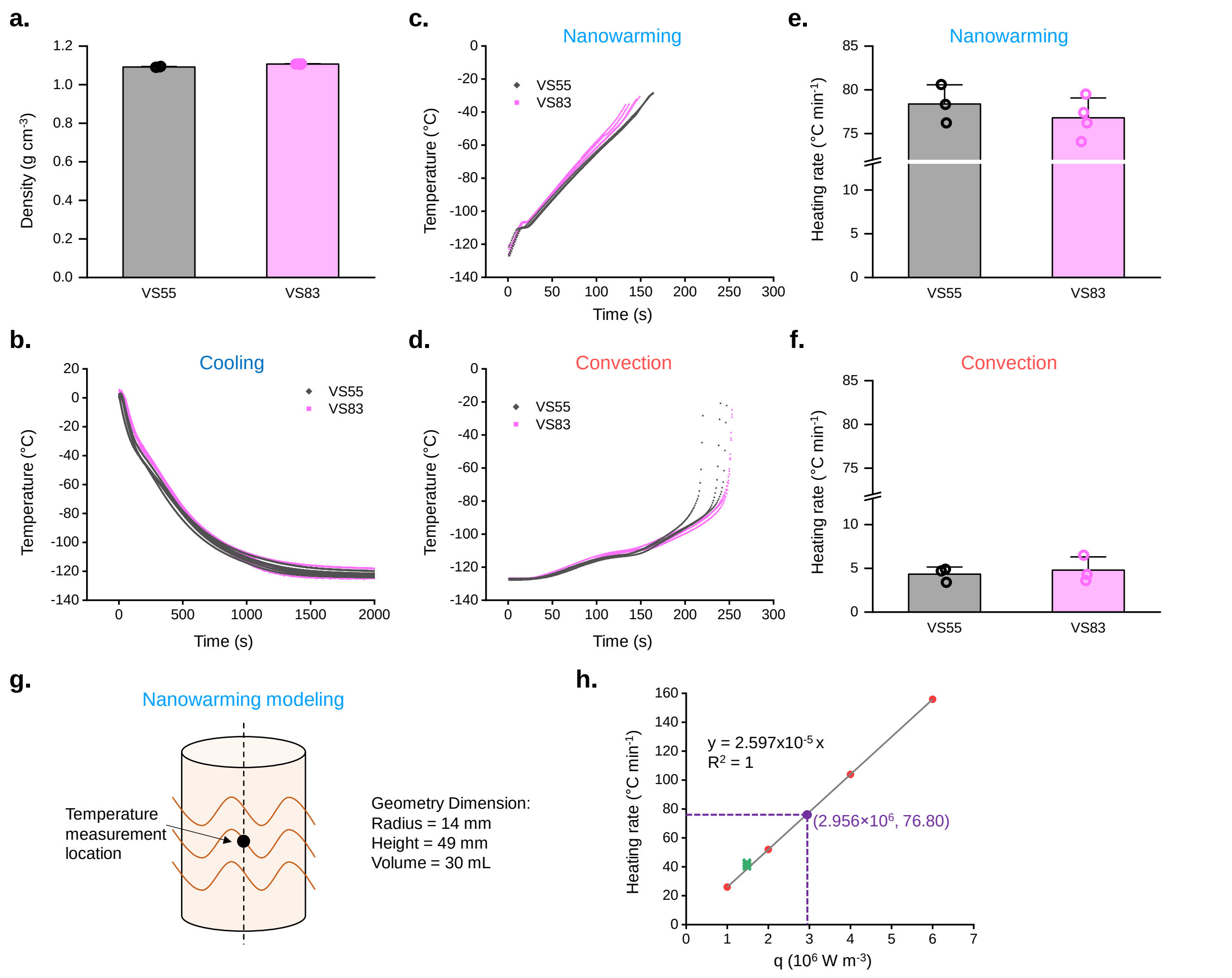

Supplementary Figure 5: VS83 thermal property similar to VS55. a Density results of VS55 and VS83 measured using the density determination kit and the analytic balance. n = 4 independent measurements for each solution. **b** Temperature profile of VS55 (n = 6 independent samples) and VS83 (n = 4 independent samples) following the same cooling process with the pure CPA solution. **c** Temperature profile of VS55 (n = 3 independent samples) and VS83 (n = 4 independent samples) solution following the same nanowarming protocol with the pure CPA solution. The mIONP concentration for VS55 and VS83 are both 2 mg ml^{-1}. **d** Temperature profile of VS55 (n = 3 independent samples) and VS83 (n = 3 independent samples) following the same convection warming process. **e** Nanowarming heating rate of VS55 (n = 3 independent samples) and VS83 (n = 4 independent samples) derived from the temperature profile in **c**. **f** Convection warming heating rate of VS55 (n = 3 independent samples) and VS83 (n = 3 independent samples) derived from the temperature profile in **d**. **g** Geometry of the CPA solution nanowarming. The tube was filled with CPA solutions with mIONPs. The dashed black line indicates the cylinder axis. The black dot shows the location for temperature measurement. **h** Heating rate results (red dots) obtained from computational modeling with different values of heat generation rate, *q*, as the input in the model. The heating rate was measured at the cylinder axial central point in the modeling (black dot shown in **g**). The grey line is the linear fitting of the modeling data points (red dots). The fitted equation and the R squared value are inserted in the

figure. The purple dot indicates the data determined through the nanowarming experiments in VS83 with 2 mg ml^{-1} mIONPs. According to the linear relationship between the heating rate and heat generation rate, we obtain the corresponding value of the heat source term, $q = 2.956 \times 10^6$ W m^{-3}, for the heating rate, 76.80 °C min^{-1}. Green cross shows the heating rate results measured from the nanowarming experiments in VS83 with 1 mg ml^{-1} mIONPs. n = 4 independent samples. All data depict mean ± standard deviation.

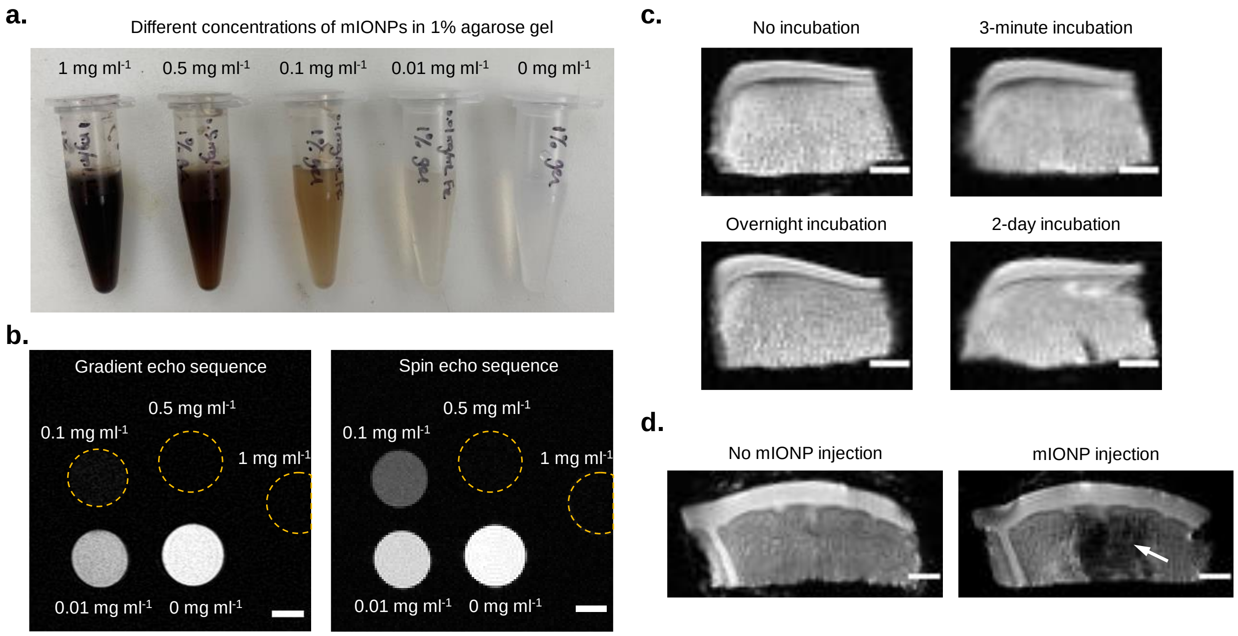

Supplementary Figure 6: Assessment of mIONPs in phantom samples and OCAs. a Photos of different concentrations of mIONPs in 1% agarose gel. **b** MRI scans of the agarose gel with different concentrations of mIONPs using gradient echo sequence and spin echo sequence. **c** MRI scans of OCA incubated in VS83 solutions with 2 mg ml^{-1} mIONPs for 0 minutes (no incubation), 3 minutes, overnight (~ 12 hours), and 2 days (~ 48 hours) using spin echo sequence. **d** MRI scans of OCA with or without mIONP injection using spin echo sequence. The white arrow highlights the injection region with diminished MRI signals due to the presence of mIONPs. All scale bars represent 4 mm.
