# Supplementary material for: Nanowarming and ice-free cryopreservation of large sized, intact porcine articular cartilage
Source: Commun Biol. 2023 Feb 24;6:220. doi: 10.1038/s42003-023-04577-9 (PMC9958003; doi:10.1038/s42003-023-04577-9)
Supplement: Supplementary file 5 — Reporting Summary [file 42003_2023_4577_MOESM5_ESM.pdf]

nature portfolio

Corresponding author(s): Hai Yao

Last updated by author(s): 01/27/2023

Reporting Summary

Nature Portfolio wishes to improve the reproducibility of the work that we publish. This form provides structure for consistency and transparency in reporting. For further information on Nature Portfolio policies, see our Editorial Policies and the Editorial Policy Checklist.

Statistics

For all statistical analyses, confirm that the following items are present in the figure legend, table legend, main text, or Methods section.

n/a	Confirmed	
☐	☒	The exact sample size (*n*) for each experimental group/condition, given as a discrete number and unit of measurement
☐	☒	A statement on whether measurements were taken from distinct samples or whether the same sample was measured repeatedly
☐	☒	The statistical test(s) used AND whether they are one- or two-sided<br>*Only common tests should be described solely by name; describe more complex techniques in the Methods section.*
☐	☒	A description of all covariates tested
☐	☒	A description of any assumptions or corrections, such as tests of normality and adjustment for multiple comparisons
☐	☒	A full description of the statistical parameters including central tendency (e.g. means) or other basic estimates (e.g. regression coefficient) AND variation (e.g. standard deviation) or associated estimates of uncertainty (e.g. confidence intervals)
☐	☒	For null hypothesis testing, the test statistic (e.g. *F*, *t*, *r*) with confidence intervals, effect sizes, degrees of freedom and *P* value noted<br>*Give P values as exact values whenever suitable.*
☒	☐	For Bayesian analysis, information on the choice of priors and Markov chain Monte Carlo settings
☒	☐	For hierarchical and complex designs, identification of the appropriate level for tests and full reporting of outcomes
☒	☐	Estimates of effect sizes (e.g. Cohen's *d*, Pearson's *r*), indicating how they were calculated

Our web collection on statistics for biologists contains articles on many of the points above.

Software and code

Policy information about availability of computer code

Data collection	1. Olympus Fluoview (Version 4.2a) - Live/dead staining imaging<br>2. Olympus cellSens Standard 1.15 - H&E and Safranin O staining imaging<br>3. Bruker Biospin MRI GmbH Biospec 70/30 (Version 6.01) - MR imaging of osteochondral samples
Data analysis	1. ImageJ (Version 1.52a) - Cell viability quantification<br>2. COMSOL Multiphysics 5.4 - Modeling the nanowarming process<br>3. SPSS (Version 24.0) - Statistical analysis<br>4. Origin 2018 - Mechanical data analysis and generating all of the bar and scatter graphs

For manuscripts utilizing custom algorithms or software that are central to the research but not yet described in published literature, software must be made available to editors and reviewers. We strongly encourage code deposition in a community repository (e.g. GitHub). See the Nature Portfolio guidelines for submitting code & software for further information.

Data

Policy information about availability of data

All manuscripts must include a data availability statement. This statement should provide the following information, where applicable:

- Accession codes, unique identifiers, or web links for publicly available datasets
- A description of any restrictions on data availability
- For clinical datasets or third party data, please ensure that the statement adheres to our policy

All resources and raw data are available from the authors upon reasonable request.

Human research participants

Policy information about studies involving human research participants and Sex and Gender in Research.

Reporting on sex and gender	NA
Population characteristics	NA
Recruitment	NA
Ethics oversight	NA

Note that full information on the approval of the study protocol must also be provided in the manuscript.

Field-specific reporting

Please select the one below that is the best fit for your research. If you are not sure, read the appropriate sections before making your selection.

☒ Life sciences ☐ Behavioural & social sciences ☐ Ecological, evolutionary & environmental sciences

For a reference copy of the document with all sections, see nature.com/documents/nr-reporting-summary-flat.pdf

Life sciences study design

All studies must disclose on these points even when the disclosure is negative.

Sample size	Sample sizes were estimated based on our previously published tissue preservation and tissue characterization studies (Brockbank, et al. 2011; Hepfer, et al. 2018; Wu, et al. 2015). The sample size of each experiment was stated in the figure caption.
Data exclusions	No data was excluded.
Replication	All measurements were successfully repeated in at least 3 independent samples.
Randomization	Porcine osteochondral samples were randomly allocated to different groups.
Blinding	The investigators were blinded to the group allocation during data collection and analysis.

Reporting for specific materials, systems and methods

We require information from authors about some types of materials, experimental systems and methods used in many studies. Here, indicate whether each material, system or method listed is relevant to your study. If you are not sure if a list item applies to your research, read the appropriate section before selecting a response.

Materials & experimental systems

n/a	Involved in the study
☒	☐ Antibodies
☒	☐ Eukaryotic cell lines
☒	☐ Palaeontology and archaeology
☐	☒ Animals and other organisms
☒	☐ Clinical data
☒	☐ Dual use research of concern

Methods

n/a	Involved in the study
☒	☐ ChIP-seq
☒	☐ Flow cytometry
☒	☐ MRI-based neuroimaging

Animals and other research organisms

Policy information about studies involving animals; ARRIVE guidelines recommended for reporting animal research, and Sex and Gender in Research

Laboratory animals	4-6 months old Yorkshire pigs
Wild animals	This study did not involve wild animals
Reporting on sex	Not applicable. The animals used in this study have a mixed sex (with both male and female). We intend to demonstrate the general application of this new preservation method on cartilage preservation by applying a mixed sex in this study. No sex analysis were performed for this initial phase study. More detailed analysis could be performed in the future study.
Field-collected samples	This study did not involve samples collected from the field
Ethics oversight	The use of animal tissues was approved by Clemson University and Medical University of South Carolina. Porcine knees were obtained from a local abattoir. No animals were specifically sacrificed for this study.

Note that full information on the approval of the study protocol must also be provided in the manuscript.
